# Books

**Published:** 1882-08

**Authors:** 


### Books.

THE PHYSICIAN HIMSELF AND WHAT HE SHOULD ADD TO THE STRICTLY SCIENTIFIC. By D. W. Cathell, M.D. Baltimore: Cushings & Bailey. 1882. Pp. 188. Price, $1.25.

The author has combined and embodied in an attractive form, the substance of many disconnected pencil-notes, made at odd moments, during an active professional life, setting forth his ideas concerning the proper deportment and the personal conduct of medical practitioners. The tone of the book and the evident intent of the writer is good. There is undoubtedly a great deal of worldly wisdom in its pages. Some of the advice offered will appear to the majority of readers trite, and seem unnecessary; but as the book is intended, of course, for no exclusive class, it will probably encounter many readers who will not be unfavorably impressed by this possible defect. The suggestions referring to the importance of observing business principles in business transactions are excellent. Bills for professional services should be presented promptly and the payment of full fees, based upon an equitable and just standard, should be demanded.

We can scarcely approve of the style adopted by the author; the impression produced by reading the book would be more agreeable if another mode of expression had been employed. The failure to better classify the contents and to mark off the several subjects by divisions into chapters and departments seriously detracts from the value of the book and the pleasure of its perusal, and if we were disposed to be very critical we might dissent from expressions and bits of advice found here and there among its pages; but we honestly think the work is calculated to do good and that it is entitled to a cordial reception. For ourselves, however, we believe that all questions of this sort can be effectually disposed of by a conscientious observance of the golden rule—Do unto others as you would have them do unto you; remembering always, under all circumstances and in all conditions that honesty—pure and simple—is the best policy.

The type is clear and large; the paper, binding and general make up, excellent.

---

THE VEST-POCKET ANATOMIST, FOUNDED UPON GRAY. By C. Henri Leonard, A.M., M.D. Eleventh Revised Edition. Pp. 82. From C. Henri Leonard, M.D , Detroit. Price, 75 cents.

This little book, intended as a dissecting room companion and students' aid, is, in the main, a condensation from well-known anatomical and surgical works. It contains in a very concise and convenient form much important and useful, yea essential, information for the student, physician and surgeon. The fact that the modest little volume has reached its eleventh edition is conclusive evidence of popular appreciation.

The work of the publisher has been well done. The printing and binding is all that could be desired. Persons in need of such a remembrancer will find in this little candidate for their favor complete satisfaction.

---

Practical Surgical Anatomy. A guide to the physician in the study of the relations of the Viscera to each other in health and disease, and in the diagnosis of the medical and surgical conditions of the anatomical structures of the head and trunk. By Ambrose L. Ranney, A.M., M.D. New York: William Wood & Company. 1882. Wood's Library of Standard Medical Authors.

The comprehensive title of this work sufficiently explains its character and scope. It may be described as a medico-surgical anatomical compendium. The author does not claim for it consideration as a systematic "treatise upon descriptive anatomy, but rather a collection of hints which shall serve as a guide to the study of the practical bearings of the different portions of the head and trunk upon the field of general medicine."

The book is indirectly the outgrowth of two courses of lectures delivered by the writer in the medical department of the University of New York. It is generously illustrated, for the most part, with familiar cuts, and the publishers work is up to the usual standard of this well-known house.

---

Atlas of Gynæcology and Obstetrics. By Dr. E. Martin and Dr. J. P. Maygrier, containing 475 plain and 35 colored illustrations, from the original designs of many distinguished authors, with explanatory text translated and edited, with additions, by William A. Rothacker, M.D. A. E. Wilde & Company, publishers, Cincinnati, Ohio.

This artistic work is presented to the medical profession as a hand book intended to be supplementary to existing treatises on obstetrics and diseases of women. The atlas was issued in fifteen parts—each part containing four elegant plates and four pages of explanatory text—by the enterprising publishers, and now the work is complete.

Every teacher, practitioner and student must appreciate the value of, and the help derived from correct representations by accurately executed illustrations in the study of many subjects, and in no department more than in that to which the present work is devoted.

The Atlas contains over five hundred beautiful figures, thirty-five of which are colored, and all of which are skillfully fashioned and artistically finished.

The explanatory notes are models of clearness and conciseness. No claim is laid to originality in these, but "the writer has endeavored by diligent study of the modern authorities to give the main points treated in the plates correctly." The text is conveniently placed on the page opposite the plate to which it relates.

A single, momentary glance at some of these handsome engravings will produce a more lasting impression than will often be obtained by a tedious study for hours of the ordinary descriptive text-books.

The whole work reflects great credit upon the editor and the publishers, and it will not prove disappointing even to those whose expectations are fixed at the highest pitch.

